# Reply: Surgery *vs* chemoRT for locally advanced operable head and neck cancers

**DOI:** 10.1038/sj.bjc.6603077

**Published:** 2006-05-16

**Authors:** K C Soo, J Wee, E H Tan

**Affiliations:** 1 Department of Surgical Oncology, National Cancer Centre Singapore, 11 Hospital Drive, 169610 Singapore; 2 Department of Radiation Oncology, National Cancer Centre Singapore, 11 Hospital Drive, 169610 Singapore; 3 Department of Medical Oncology, National Cancer Centre Singapore, 11 Hospital Drive, 169610 Singapore

**Sir,**

We thank Schaffer *et al* for the interest in our article. They have highlighted relevant points for discussion from our study.

The conclusion of our study was that there was no statistical difference in survival between the two arms comparing surgery with postoperative radiotherapy (RT) as adjuvant and concurrent chemoradiotherapy with surgical salvage. It was a very difficult trial to undertake but an important one because up until then, there has not been such a study. For many oncologists, surgery with adjuvant radiotherapy is the standard practice for advanced resectable head and neck cancers.

The reason we chose 66 Gy instead of 70 Gy was the reported toxicity with concurrent chemoradiotherapy. It was described as ‘formidable’ (Adelstein *et al*, 1997). There were other significant institutions that used 66 Gy for definitive radiation for stage III and IV head and neck cancers (Grau *et al*, 2003), more so when chemotherapy was given concurrently. In the Adelstein/Intergroup trial (Adelstein *et al*, 2003) quoted, the RT dose of 70 Gy was given continuously only for the arm that received single agent concurrent Cisplatin. The arm that received Cisplatin and 5FU had a split course RT regimen. A better comparison would be Adelstein's previous Phase III trial (Adelstein *et al*, 1997) that treated patients to 68–72 Gy at 1.8–2 Gy fractions (even in patients with RT alone), and Cisplatin and 5FU was given on days 1 and 22 of radiotherapy. Currently, 66 Gy is still an acceptable standard (Dobbs *et al*, 1985, 1992, 1999) for radical RT for head and neck cancers. Whether there will be any relevant clinical difference between 66, 68 or 70 Gy is debatable – certainly in cancers of the oeosophagus, a randomized trial (Minsky *et al*, 2002) has failed to show any difference between 50.4 and 64.8 Gy, when Cisplatin and 5FU was given concurrently with RT. The concern in our protocol was obviously normal tissue toxicity, especially when we were adding two drugs concurrent with the RT regimen. Rather than the higher toxicity being contributed by treatment interruptions, it was precisely because of higher toxicity that there were treatment interruptions.

The low accrual rate was not because patients preferred surgery. In our context, the majority of patients preferred the nonsurgical arm and that was an important contributing factor to the low accrual.

All patients were initially assessed by the head and neck surgeon for resectability. They were then presented at a multidisciplinary tumour board, where there were in addition to radiation and medical oncologists, other surgical oncologists who provided another assessment of resectabilility.

We did not have second thoughts about whether every resectable tumour is preferably treated by surgery rather than chemoradiotherapy. We were reporting what we observed at the end of the study.

As to the question, which T_4_ tumours should be operated, it will be difficult to generalize. What we can say is that there is no difference in survival with either therapy and other considerations will need to play a role in the decision making. While we agree that in a significant number of patients with laryngeal cancer, the disease can be managed without surgery, it cannot be for all. The EOTRC (Bernier *et al*, 2004) and the RTOG (Cooper *et al*, 2004) postoperative trials had significant number of patients with laryngeal cancers. There are, notwithstanding the VA study of 1991 (The Department of Veterans Affairs Laryngeal Cancer Study Group, 1991), considerable number of oncologists who can ethically and with a clear conscience refer patients for definitive surgical treatment of advanced laryngeal cancers.
